# Quantitative analysis of mitral valve morphology in atrial functional mitral regurgitation using real-time 3-dimensional echocardiography atrial functional mitral regurgitation

**DOI:** 10.1186/s12947-018-0131-1

**Published:** 2018-08-21

**Authors:** Tao Cong, Jinping Gu, Alex Pui-Wai Lee, Zhijuan Shang, Yinghui Sun, Qiaobing Sun, Hong Wei, Na Chen, Siyao Sun, Tingting Fu

**Affiliations:** 1 Department of Cardiology, The First Affiliated Hospital of Dalian Medical University, Dalian, 116000 Liaoning China; 2 Department of Intensive Care Unit, The Second Affiliated Hospital of Dalian Medical University, Liaoning, China; 3 Division of Cardiology, Department of Medicine and Therapeutics, The Prince of Wales Hospital of Chinese University of Hong Kong, Hong Kong, China

**Keywords:** Atrial fibrillation, Mitral regurgitation, Three-dimensional transesophageal echocardiography

## Abstract

**Background:**

Atrial fibrillation (AF) can result in atrial functional mitral regurgitation (MR), but the mechanism remains controversial. Few data about the relationship between the 3-dimensional morphology of the MV and the degree of MR in AF exist.

**Methods:**

Real-time 3-dimensional transesophageal echocardiography (3D-TEE) of the MV was acquired in 168 patients with AF (57.7% persistent AF), including 25 (14.9%) patients with moderate to severe MR (the MR+ group) and 25 patients without AF as controls. The 3-dimensional geometry of the MV apparatus was acquired using dedicated quantification software.

**Results:**

Compared with the group of patients with no or mild MR (the MR- group) and the controls, the MR+ group had a larger left atrium (LA), a more dilated mitral annulus (MA), a reduced annular height to commissural width ratio (AHCWR), indicating flattening of the annular saddle shape, and greater leaflet surfaces and tethering. MR severity was correlated with the MA area (r^2^ = 0.43, *P* < 0.01) and the annulus circumference (r^2^ = 0.38, P < 0.01). A logistic regression analysis indicated that the MA area (OR: 1.02, 95% CI: 1.01–1.03, P < 0.01), AHCWR (OR: 0.24, 95% CI: 0.14–0.35, *P* = 0.04) and MV tenting volume (OR: 3.24, 95% CI: 1.16–9.08, *P* = 0.03) were independent predictors of MR severity in AF patients.

**Conclusions:**

The mechanisms of “atrial functional MR” are complex and include dilation of the MA, flattening of the annular saddle shape and greater leaflet tethering.

## Background

The MV apparatus consists of several components: the LA, MA, mitral leaflets, chordae tendineae, papillary muscles, and left ventricle (LV). Dysfunction of any one of these components can lead to MR. MR can be classified according to the presence of mitral leaflet disease (organic MR) or only secondary involvement of the leaflets (functional MR) [1] and can also be categorized according to Carpentier’s classification as normal (Type I), excessive (Type II), or restrictive (Type III) according to the motion of the MV [2]. Among these types of MR, normal leaflet motion MR is less common than the other types and almost exclusively results from organic leaflet disease [1]. However, AF may lead to Type I MR, even though the mechanism remains controversial. Most reports have suggested that AF causes functional Type I MR through atrial remodeling that leads to MA dilation [3–5]. However, it cannot be determined at present whether combinations of other structural MV abnormalities occur in AF patients with MR (e.g., changes in MA geometry or the size of the MV leaflets). An understanding of these mechanisms may provide more detailed information for surgeons for planning the surgical treatment of AF in patients with severe MR [6].

With the development of 3D-TEE, high-resolution imaging and quantification of the morphology of the entire mitral apparatus have become feasible [7–10]. 3D-TEE studies of MV geometry have led to a new understanding of the pathogenesis of functional MR. [11, 12] Few data exist on the relationship between the 3D morphology of the MV and the degree of MR in AF, which is a common cause of functional MR. [3, 13] Therefore, we undertook a 3D-TEE study in patients with AF to investigate the relationship between 3D MV morphology and clinically moderate to severe MR.

## Methods

### Patient selection

**Fig. 1:**
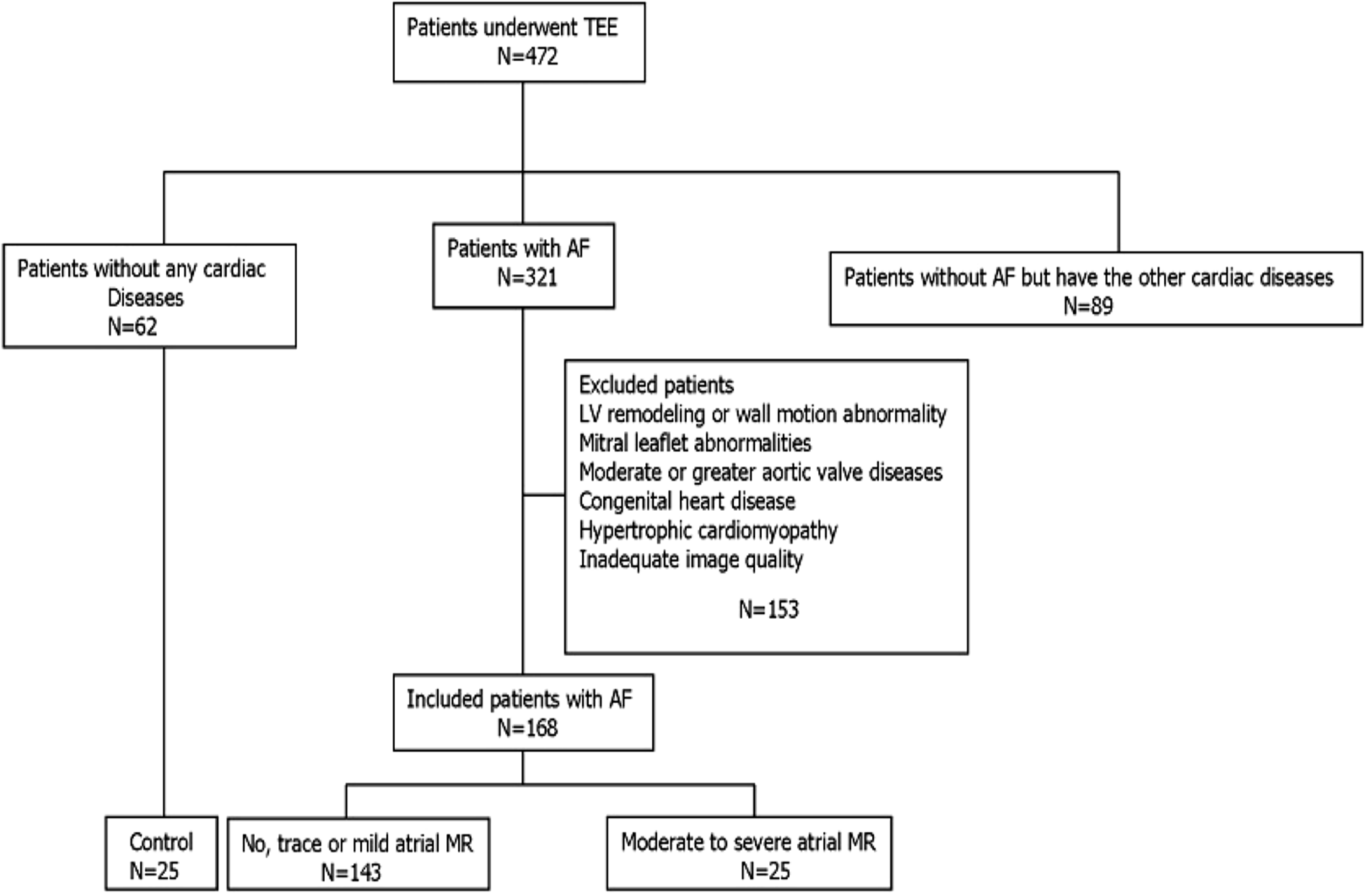
Flow diagram of patients included in the analysis. AF, atrial fibrillation; MR, mitral regurgitation; TEE, transesophageal echocardiography

We retrospectively screened 321 patients with AF out of 472 patients who underwent transthoracic echocardiography (TTE) and 3D-TEE on the same day due to clinical indications between June 2014 and May 2016 (Fig. 1). The study population consisted of consecutive patients with symptom improvement or drug-refractory AF, including persistent or paroxysmal AF,they all fit the indications of ablation according to the guideline [14].We performed 3D-TEE to exclude left atrial thrombi and collected images of MR and 3D images of the MV. AF patients were excluded from the study if they had organic heart disease (including rheumatic heart disease, coronary heart disease, congenital heart disease, MV prolapse, moderate to severe MA or MV calcification according to the echocardiographic calcification score [15] or MV surgery, etc.), Patients with a left ventricular ejection fraction (LVEF) < 50% were excluded to avoid including patients whose MR might be due to ventricular dysfunction. AF was classified according to the clinical characteristics into two types: paroxysmal AF (self-terminating, lasting for < 7 days) and persistent AF (lasting ≥7 days or terminated by intervention). We enrolled sex-, age-, and body surface area-matched patients with no AF and MR from the remaining cohort.

### Echocardiography

At first, using TTE, we obtained routine parameters, including left ventricular end diastolic diameter (LVEDD), left ventricular end systolic diameter (LVESD) and left atrial anterior posterior diameter (LAAPD), using an iE Elite system (Philips Healthcare, Andover, MA, USA). The LV volume and LVEF were calculated using a modified version of Simpson’s method. LA volume was defined as the largest LA volume just before the MV opening and was measured from an apical view using the biplane method of discs. Diastolic function was described with in terms of the E/E′ ratio, where E was measured using pulsed Doppler of the mitral inflow from the four-chamber view, and E′ was the average of the septal and lateral MA diastolic velocities using tissue Doppler imaging. For the patients with AF, the above data were measured during an index beat, which was the beat after the nearly equal preceding and prepreceding intervals [16]. 3D-TEE of the MV was performed using a fully sampled matrix transducer (X7-2 t). Zoomed 3D images of the mitral apparatus, including the annulus, leaflets, and aortic valve were acquired. The region of interest was adjusted to the smallest volume to obtain higher frame rates (> 8). For patients with sinus rhythm, the 3D images were recorded with multiple cardiac beats (2–4 beats), and for patients with AF, 3D data were acquired from the index beat, as described above. The images were acquired carefully to ensure optimal image quality without stitching artifacts. Under the color Doppler model, the maximum regurgitation jet area (RJA) and the effective regurgitation orifice (ERO) were obtained, and the ERO was used to assess the severity of MR [17, 18]. The MR color jet area was measured based on mosaic signals in the apical 4-chamber, apical 2-chamber, and apical long-axis views, and the color Doppler scale and Nyquist limit were set to 50–70 cm/s. The ERO of the MR was quantified using the proximal isovelocity surface area method [19]. Moderate to severe MR was defined by the presence of following criteria: ERO ≥ 0.3 cm^2^ (MR+). Mild MR was defined by the presence of the following criteria: ERO < 0.3 cm^2^ (MR-). In addition, supportive parameters, including RJA, continuous wave Doppler jet configuration, pulsed wave Doppler transmitral flow, and pulmonary venous flow were examined in an integrative approach for evaluating MR severity, as previously recommended [19]. In addition, the patients with no or trace MR for whom the ERO could not be obtained were also included in the MR- group.

### Quantitative assessment of the mitral apparatus

**Fig. 2:**
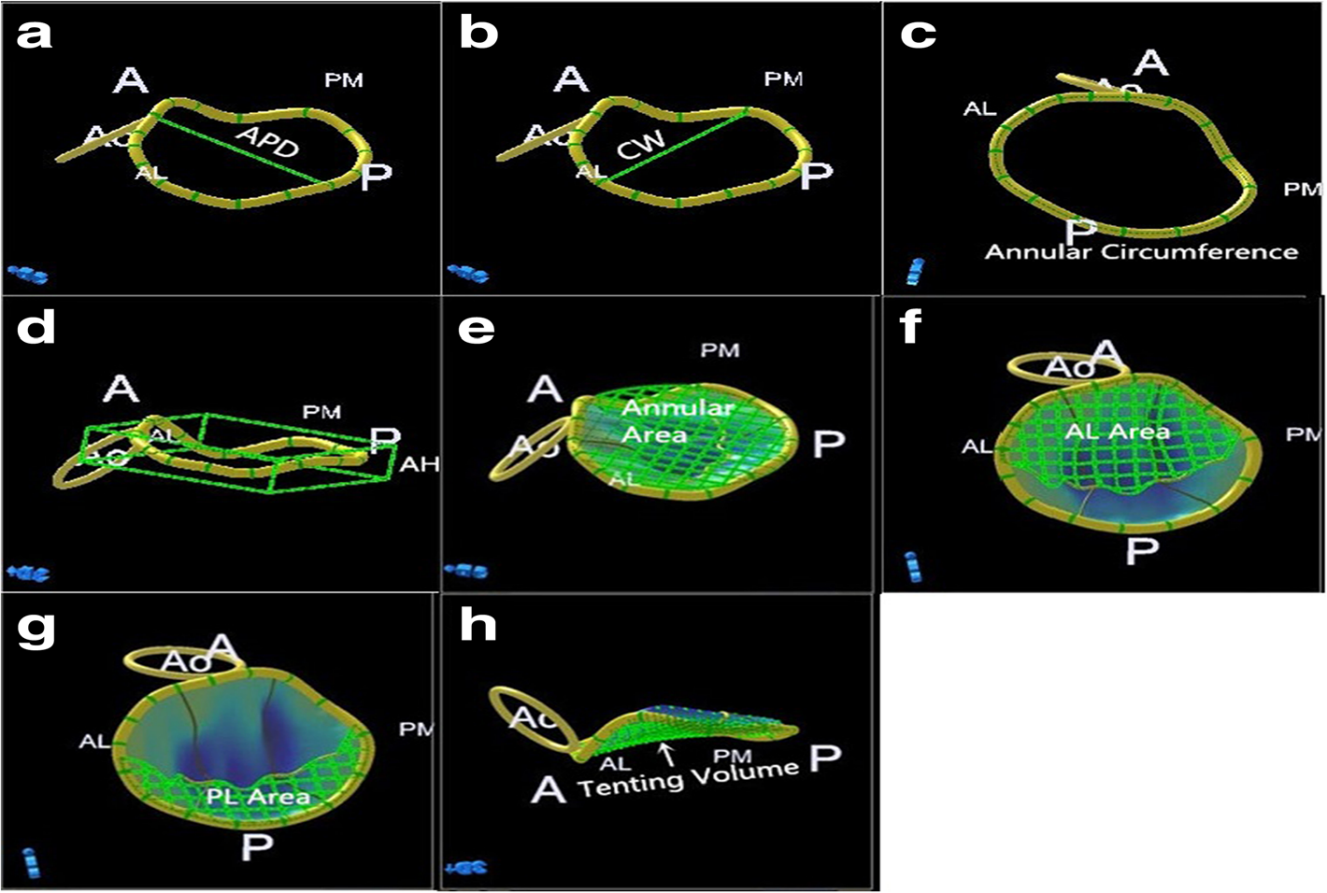
Parameters of 3-dimensional geometry of the mitral valve. A, Anteroposterior diameter (APD). B, Commissural width (CW). C, Annular circumference. D, Annular height (AH). E, Annular area on the projection plane. F and G, Areas of the exposed anterior leaflet (AL) and posterior leafet (PL) surfaces. H, Leaflet tenting volume

Images were analyzed offline by an investigator. The quantitative morphological analysis of the mitral valve was performed using dedicated software (QLAB MVQ, Philips 9.1 version) [20]. The images were presented in 4 quadrants, including 3 orthogonal planes, each representing an anatomic plane derived from the 3D data, and a volume-rendered view. The end-systolic frame, immediately before aortic valve closure, was tagged in the cine-loop sequence. The image was oriented by adjusting the rotation of the image data in the orthogonal planes thus ensuring that the mitral valve was bisected by the 2 long-axis planes and that the short-axis plane was parallel to the plane of the valve. Initially, the 4 major annulus reference points were tagged in the appropriate planes. The annulus shape was then manually outlined by defining intermediate reference points in 18 radial planes (i.e., 36 reference points) that were rotated around the long axis. The mitral valve was then segmented to map the leaflet contour and coaptation by manually tracing the leaflets in multiple parallel long-axis planes spanning the valve from commissure to commissure (6 trace points per centimeter). Finally, the reconstructed mitral valve apparatus was displayed, and the parameters were automatically generated. Parameters describing the annular geometry included anteroposterior diameter (APD), commissural width (the distance between the posteromedial and anterolateral horns of the annulus, CW), height (the maximal vertical distance between the highest and lowest annular points AH), projected area (AA) and circumference (AC). The ratio of annular height-to-commissural width (AHCWR) was computed as an indicator of annular saddle-shaped nonplanarity. We studied the 3D leaflet surface topography by assessing the leaflet area [including anterior leafet (AL) and posterior leafet (PL) area]and tenting volume (Fig. 2).

### Statistical analysis

Continuous variables are expressed as the mean ± SD, and categorical variables are expressed as absolute values and percentages. Normal distribution of the continuous parameters was verified using the Kolmogorov–Smirnov test and compared using one-way analysis of variance (ANOVA) with post-test Bonferroni correction. Categorical variables were compared using the Chi-squared test or Fisher’s exact test where indicated. All tests were two-sided, and statistical significance was defined as *P* < 0.05. Correlations between continuous variables were explored using Pearson analysis. Variables with a *P*-value less than 0.1 between patients of the MR+ and MR- groups were included in a multivariable logistic regression analysis using stepwise forward elimination to identify factors with independent associations with the severity of MR (duration of AF, LA volume, E/E’, annulus area, annulus circumference, annulus CW, AHCWR, AL surface area, PL surface area and leaflet tenting volume). To evaluate the reliability of 3D echocardiographic results, intra-observer and inter-observer variability was assessed. 15 subjects were randomly chosen for that analysis, The intraclass correlation coefficient (ICC) was calculated.

## Results

### Study population and baseline characteristics

Complete datasets were available for 168 patients with AF (paroxysmal AF: 95, 56.5%). We divided the patients into 2 groups according to the severity of MR (MR+ and MR- groups). Twenty-five patients with moderate to severe MR according to the standard were included in the MR+ group, while other patients with no, trace or mild regurgitation were included in the MR- group. Twenty-five patients with no AF and MR were included in the controls. The clinical characteristics of the three groups are listed in Table 1. Patients in the MR+ group, when compared to those in the MR- group, had a longer duration of AF (4.9 ± 0.5 VS 4.3 ± 1.0 years, *P* <0.01), and there was no significant difference in the types of AF between the two groups (paroxysmal AF: 12 [48.8%] vs. 83 [58.2%], *P* = 0.12).

**Table 1:** Baseline characteristics

variable	Control (*n* = 25)	Patients with AF (*n* = 168)		*P*
		MR- Group (143)	MR+ Group (n = 25)	
Age, years	66 ± 7	62 ± 6	66 ± 7	0.20
Women, n (%)	12(48.9)	68(47.8)	11(46.9)	0.96
Body surface area, m^2^	1.88 ± 0.16	1.92 ± 0.18	1.87 ± 0.15	0.22
Paroxysmal AF, n (%)		83(58.2%)	12(48.8%)	0.12
Duration of AF, years		4.3 ± 1.0	4.9 ± 0.5^#^	<0.01
History of Hypertension, n (%)	14 (56%)	90 (58%)	16 (64%)	0.55
History of Diabetes Mellitus, n (%)	5 (20%)	32 (21%)	4 (16%)	0.6
Status during echocardiography				
Sinus rhythm, n (%)	25(100%)	36(25.1%)*	8(30.4%)*	<0.01
Heart rate, (beats/min)	84 ± 17	89 ± 16	92 ± 12	0.46
Systolic blood pressure, mmHg	128 ± 13	134 ± 12	132 ± 16	0.86
Diastolic blood pressure, mmHg	68 ± 9	77 ± 10	73 ± 8	0.59

*P <0.01 vs. controls, #P<0.01 vs. MR-Group

### TTE findings

The TTE measurements are shown in Table 2. As expected by design, in the MR+ group, patients had increased LA volume compared with patients in the MR- group and controls (MR+ 98 ± 22 ml vs. MR- 81 ± 18 ml, *P* < 0.01; both *P* < 0.01 vs. controls 57 ± 5 ml). The E/E′ ratio was significantly elevated in the MR+ group, whereas it was comparable in the MR- group and controls (MR+ 9.2 ± 1.2 vs. MR- 8.2 ± 0.8, *P* < 0.01; MR+ vs. controls 8.0 ± 0.9, *P* < 0.01). The ERO of the MR+ group was also larger than that of the MR- group (0.47 ± 0.07 cm^2^ vs. 0.18 ± 0.07 cm^2^, *P* < 0.01).

**Table 2:** Measurements on Transthoracic Echocardiography

variable	Control (n = 25)	Patients with AF (n = 168)		*P*
		MR- Group (143)	MR+ Group (n = 25)	
LV end-diastolic diameter, mm	44 ± 2	46 ± 4	46 ± 3	0.17
LV end-systolic diameter, mm	30 ± 2	30 ± 3	31 ± 3	0.31
LV end-diastolic volume, ml	85 ± 8	87 ± 11	89 ± 12	0.20
LV end-systolic volume, ml	30 ± 4	32 ± 5	31 ± 5	0.30
LV ejection fraction, %	64 ± 5	64 ± 4	64 ± 2	0.66
LA anterior-posterior Diameter, mm	34 ± 2	39 ± 5*	41 ± 4*	<0.01
LA volume, ml	57 ± 5	81 ± 18*	98 ± 22*^,#^	<0.01
E/E’	8.0 ± 0.9	8.2 ± 0.8	9.2 ± 1.2*^#^	<0.01
Mitral regurgitation severity				
Regurgitation area, cm^2^		1.84 ± 0.52	4.37 ± 0.68^#^	<0.01
EROA, cm^2^		0.18 ± 0.07	0.47 ± 0.07^#^	<0.01

*LV* left ventricle, *LA* left atrium, *EROA* effective regurgitant orifice area. *P <0.01 vs. controls, #P<0.01 vs. MR-Group

### 3D mitral valve geometry

#### Annulus

Compared with those in the MR- group and controls, patients in the MR+ group had A dilated MA with a significantly increased annulus area, circumference and commissural width. Commonly, the mitral annulus adopts a non-planar saddle shape [21–23] with elevation of the anterior and posterior annular segments, and the low points of the saddle are close to the lateral and medial commissures. In this study, the average AHCWR value of patients in the MR+ group was significantly lower than that of patients in the MR- group and controls (MR+ 15 ± 5% vs. MR- 17 ± 4%, *P* < 0.05; both P < 0.05 vs. controls 19.4 ± 4.3%), indicating the progressive flattening of the mitral annulus (Table 3) in patients with AF with moderate to severe MR.

**Table 3:** Three-Dimensional Mitral Valve Geometry

variable	Control (n = 25)	Patients with AF (n = 168)		*P*
		MR- Group (143)	MR+ Group (*n* = 25)	
Annulus				
Area cm^2^	8.86 ± 1.03	9.53 ± 1.42*	12.66 ± 0.85*^#^	<0.01
Circumference mm	108 ± 9	115 ± 8*	134 ± 13*^#^	<0.01
Anteroposterior diameter mm	30.6 ± 2.4	32.0 ± 20.4	32.9 ± 20.4	0.90
Commissural width mm	36.3 ± 2.3	38.3 ± 3.0*	41.0 ± 3.8*^#^	<0.01
High mm	7.6 ± 2.0	6.3 ± 1.5*	6.2 ± 2.2*	<0.01
AHCWR %	19.4 ± 4.3	17.3 ± 4.4^†^	15.2 ± 5.1*^‡^	<0.01
Leaflet				
Anterior leaflet surface area cm^2^	5.38 ± 0.99	5.34 ± 1.81	6.81 ± 1.91*^#^	<0.01
Posterior leaflet surface area cm^2^	5.96 ± 1.37	5.41 ± 1.49	6.14 ± 1.30^‡^	0.03
MV tenting volume ml	1.72 ± 0.73	1.72 ± 0.89	2.67 ± 1.07*^#^	<0.01

*AHCWR* annular height-to-commissural width ratio, *MV* mitral valve

†*P* <0.05 vs. controls, ‡*P* <0.05 vs. MR-Group. *P <0.01 vs. controls, ^#^*P* <0.01 vs. MR-Group

#### Leaflet

**Fig. 3:**
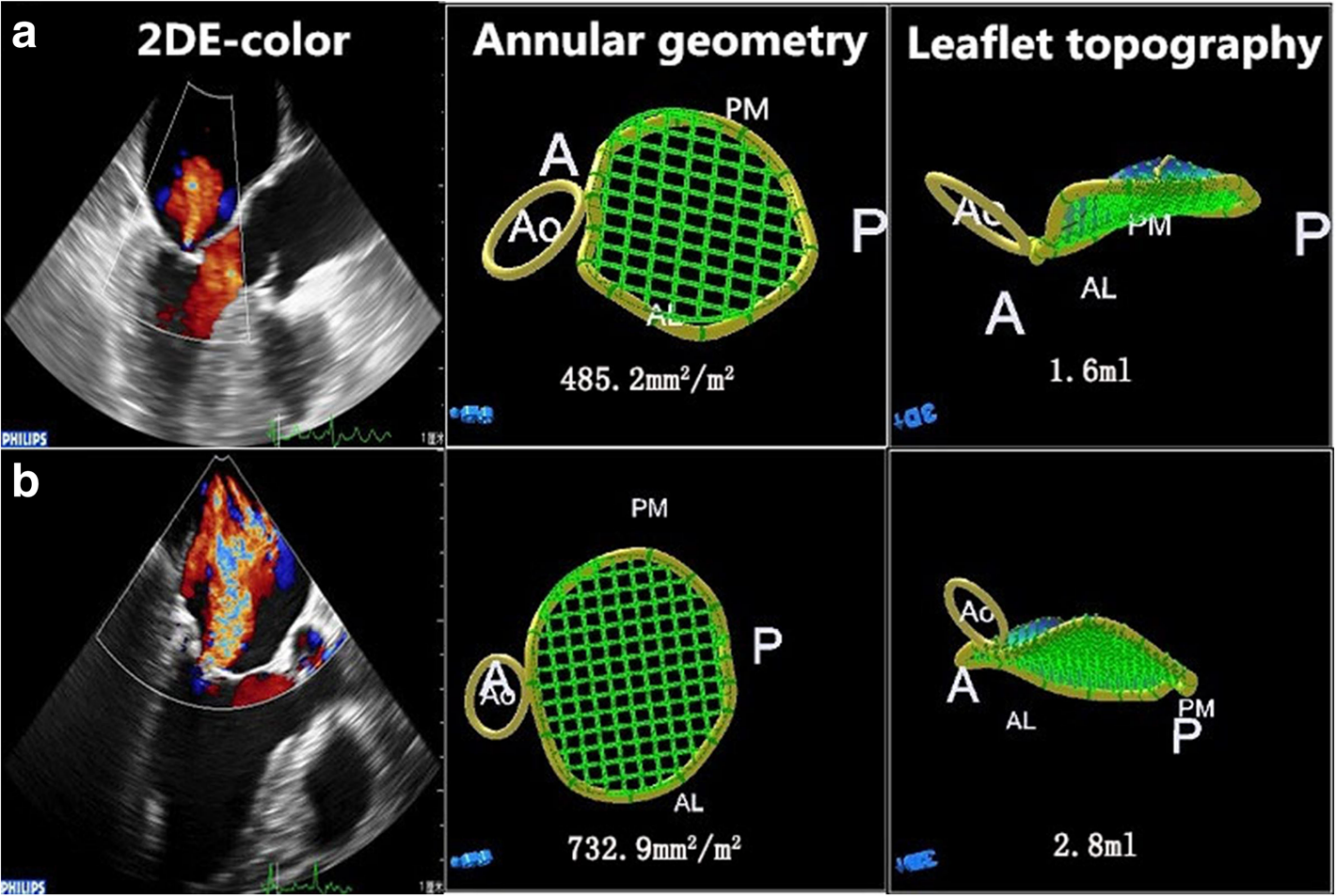
Differences in the MV morphology between mild and severe atrial functional MR in patients with AF. A, Example of mild atrial MR with the annular area and leaflet tenting volume. B, Example of severe atrial functional MR with enlargement of the annular area and leaflet tenting volume

Compared with those in the MR- group and controls, the leaflet surface area (anterior: MR+ 6.81 ± 1.91 cm^2^ vs. MR- 5.34 ± 1.81 cm^2^ *P* < 0.01, MR+ vs. controls 5.38 ± 0.99 cm^2^, *P* < 0.01; posterior: MR+ 6.14 ± 1.30 cm^2^ vs. MR- 5.41 ± 1.49 cm^2^, *P* = 0.02) and tenting volume of the mitral valve (MR+ 2.67 ± 1.07 ml vs. MR- 1.72 ± 0.89 ml, *P* < 0.01, MR+ vs. controls 1.72 ± 0.73 ml, *P* < 0.01) were significantly higher in patients in the MR+ group (Fig. 3).

### 3D mitral valve morphology is associated with MR severity

**Fig. 4:**
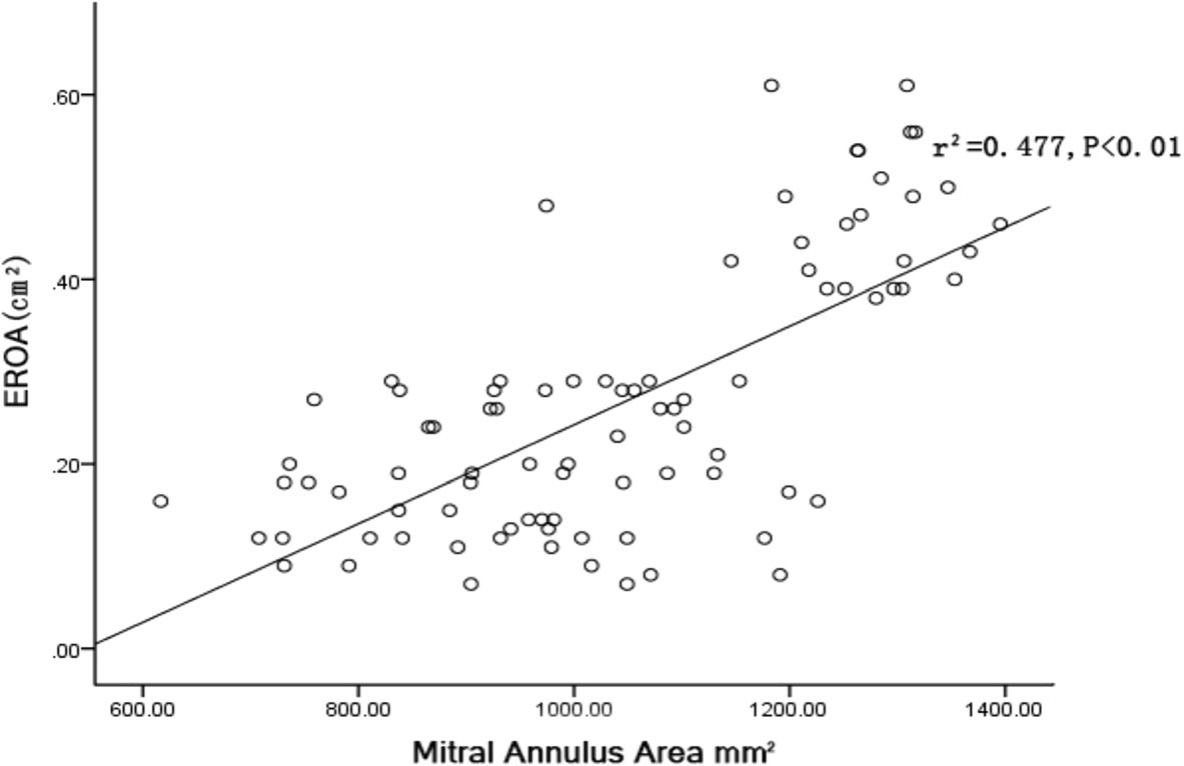
Correlation of the effective regurgitation orifice area (EROA) with the mitral annulus area

**Fig. 5:**
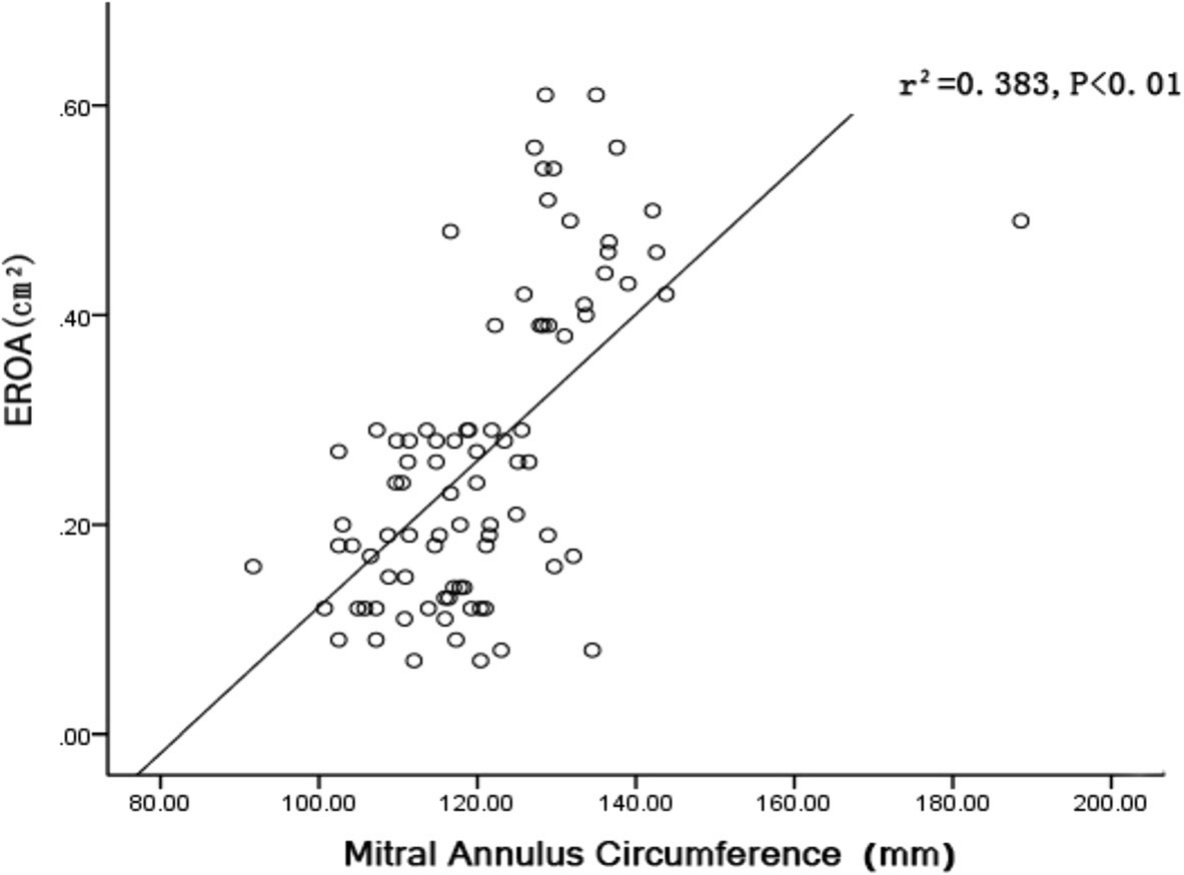
Correlation of the effective regurgitation orifice area (EROA) with the mitral annulus circumference

Structural deformation of the mitral valve increased with increasing MR severity in patients with AF. Larger EROs were found to be associated with greater annular area (r^2^ = 0.48, *P* < 0.01; Fig. 4) and annular circumference (r^2^ = 0.38, *P* < 0.01; Fig. 5).

To identify independent factors associated with MR severity in patients with AF, stepwise logistic regression analysis was performed; variables found to be significant or of borderline significance by univariate analysis (*P* ≤ 0.10), including duration of AF, LA volume, E/E’, AA,  AC,  annulus CW, AHCWR, AL surface area,PL surface area and leaflet tenting volume, were included in the equation. The AA  (OR: 1.02, 95% CI: 1.01–1.03, *P* < 0.01), AHCWR (OR: 0.24, 95% CI: 0.14–0.35, *P* = 0.04) and MV tenting volume (OR: 3.24, 95% CI: 1.16–9.08, *P* = 0.03) were found to be independent risk factors affecting the severity of MR.

### Reproducibility of the MV parameters

Intraobserver ICC for AA, AC, APD, CW and AH were 0.91 (95% CI, 69–98%), 0.84 (95% CI, 46–96%), 0.88 (95% CI, 58–97%), 0.86 (95% CI, 55–96%), 0.97 (95% CI, 86–99%), Intraobserver ICC for AL surface area, PL surface area and MV tenting volume were 0.95 (95% CI, 81–99%), 0.87 (95% CI, 57–97%), 0.91 (95% CI, 70–98%). Interobserver ICC for AA, AC, APD, CW and AH were 0.79 (95% CI, 34–94%), 0.90 (95% CI, 64–98%), 0.92 (95% CI, 71–98%), 0.86 (95% CI, 54–96%), 0.87 (95% CI, 57–97%), Interobserver ICC for AL surface area, PL surface area and MV tenting volume were 0.81 (95% CI, 18–95%), 0.95 (95% CI, 81–99%), 0.92 (95% CI, 74–98%).

## Discussion

The results of our study indicated the following: (1) long-lasting AF including paroxysmal AF causes atrial functional MR; (2) atrial functional MR has multiple deterioration factors, including LA enlargement, morphological changes of the mitral apparatus, and MA dilatation; and (3) flattening of the annular saddle shape and leaflet tethering are independent factors for determining the severity of atrial functional MR.

In our study, 15.0% of AF patients exhibited moderate or severe MR. These results are in accordance with previous studies [3, 4, 10]. Gertz et al. reported severe MR in 6.5% of AF patients, and MR was alleviated in patients with sinus rhythm after cardioversion [3]. Another study indicated that the incidence of moderate and severe MR were higher in AF patients (66% vs 6%) [13]. However, the findings from other studies were not consistent with these results; Otsuji or Zhou et al. [24, 25] thought that isolated AF may not lead to severe MR. Combining our findings with those of previous studies, we propose that AF may be a cause of moderate to significant MR rather than simply a concurrent finding. As noted by Gertz et al., this can be called atrial functional MR.

### Pathophysiology of atrial functional MR

Using 3D-TEE, we were able to evaluate the possible pathophysiological mechanism underlying atrial functional MR. Previous studies have indicated that the mechanism of MR include LA enlargement and MA dilatation. In recent years, some scholars have studied the mechanism of atrial functional MR using 3D-TEE [12, 26, 27]; in addition to the changes described above, they also found geometric changes of the MA and the insufficient leaflet remodeling to annular dilatation. However, the subjects of the studies were patients with persistent AF, and few researchers have focused on mitral leaflet adaptations in patients with moderate to significant atrial functional MR. In our study, both paroxysmal AF and persistent AF were included to investigate whether or not paroxysmal AF could also result in atrial functional MR. In addition, although 3D-TEE was used to analyze changes in the size and MA geometrical morphology in AF patients with moderate to severe MR, we also attempted to determine the role of mitral leaflet adaptation changes in the genesis of MR.

In our study, there was no significant difference in the incidence of paroxysmal AF between the two studied groups, but the duration of AF was longer in MR+ group. Patients in the MR+ group had a larger LA, MA circumference and projection area, in addition to the observed changes in 3D MA geometry. The surrogate of annular saddle-shaped non-planarity, AHCWR, was reduced, representing a progressive flattening of the mitral annulus. This result was confirmed by another study [26], in which the author appraised the morphology and function of the MV in patients with atrial functional MR and found that the MA area, MA area fraction, nonplanarity angle, and posterior mitral leaflet angle were independent determinants of the EROA of MR. It has been postulated that the saddle shape of the annulus in systole may provide a configuration that is more capable of withstanding the stresses imposed by left ventricular pressure [11, 19], and Salgo et al. [28] demonstrated that optimal leaflet stress reductions occur with AHCWR values in the range of 15 to 20%; however, when AHCWR falls to 15%, the MA becomes more flattened, and leaflet stress increases markedly. In our study, AHCWR was found to be an independent factor leading to moderate or severe MR. Therefore, we suggest that in addition to MA dilation, flattening of the mitral annulus is also involved in the mechanism of atrial functional MR.

Interestingly, we also found that the surface area of both mitral leaflets and valvular tenting volume were significantly greater in the MR+ group. This is the phenomenon of “valvular deformation”. The first sign is enlargement of the MV area, which can be considered mitral adaptation, and has been observed in patients with LV remodeling and MA dilatation in ischemic or dilated cardiomyopathy [11]. Our results showed that the MV may have the capacity to adapt by enlargement to some extent in response to mechanical stretching from MA dilatation caused by LA remodeling. However, if the extent of MA dilation is beyond the limits of leaflet adaptation, then leaflet coaptation will be insufficient, and atrial functional MR may occur. The second sign is MV tenting, which is often present in left ventricular dysfunction [29]. In our study, the MV tenting volume was found to be an independent factor affecting MR severity, consistent with the findings of Yiu et al. [30] that the major determinant of the ERO in functional MR was mitral deformation (i.e., systolic valvular tenting, which was measured as the valvular tenting area using 2D echocardiography); the subjects in their study were patients with left ventricular dysfunction, which was different from our study. In our study, although the systolic function of the LV was normal, dilation of the MA also resulted in an increase in the distance between the papillary muscle (PM) heads and the MA. We observed an increase in valvular tenting volume using 3D echocardiography, which illustrates the above mentioned changes. Subsequent tethering restricts systolic closure motion of the MV leaflets [31]. Tenting is characterized by insufficient systolic leaflet body displacement towards the annulus, with coaptation limited to the leaflet tips [32], resulting in functional MR.

In the present study, the E/E’ ratio was higher in the MR+ group indicating that the LA pressure was increased in patients with moderate or severe functional MR, and this may be one of the important mechanisms leading to functional MR. As with another study, the author described that elevated LA pressure was a key determinant of functional MR in both patients with preserved and reduced LVEF [33]. However, in patients with MR, the E/E’ ratio is generally increased due to increased flow across the regurgitant valve. It has been reported that LV filling pressures were not predictable by the E/E’ ratio in subjects with MR and preserved LV function [34]. Therefore, further studies are needed to confirm the value of the E/E’ ratio in patients with functional mitral regulation.

### Clinical significance

Epidemiological data show that the incidence of AF gradually increases with advancing age [35], and Gertz et al. found a 6.4% incidence rate of functional MR in patients initially presenting with AF [3]. Therefore, the prevalence of atrial functional MV will increase progressively. It is generally believed that MR instigates atrial and annular remodeling in a vicious cycle and that the latter will lead to worsening MR and eventual heart failure with disease progression [1]. Therefore, atrial functional MR will become increasingly important in the future. Our findings have important implications for understanding the mechanism of atrial functional MR and the options for treatment. The main causes of atrial functional MR are the remodeling of the left atrium and the MA. The primary purpose of treatment is to reverse the remodeling of the left atrium and the MA in order to maintain sinus rhythm. Experiments have demonstrated the effectiveness of this treatment [3]. Furthermore, because the MV tenting volume independently aggravated the atrial functional MR, repair strategies should aim to restore effective leaflet coaptation by reducing the MA area and leaflet tethering at the PM, such as by simultaneous annuloplasty and chordal cutting. Several smaller studies have shown that this treatment can improve the prognosis of patients [36, 37]. On the other hand, apically directed leaflet tethering is the predominant mechanism of MR, and the risk of recurrence of MR after undersized annuloplasty is increased [38].

### Study limitations

This was a cross-sectional study; therefore, our study suffered from the typical limitations of a cross-sectional analysis. The number of patients in the study was limited; therefore, our study should be considered a hypothesis-generating study. Our study included patients with paroxysmal and persistent AF, and although our results failed to show the different effects of the two types of fibrillation on MR, in theory, the effect of persistent AF on the atrium and mitral annulus was greater and was also confirmed by the experiment [3]. The reason for this result may be the limited numbers of patients included in our study. Due to the older version of the analysis software used, we failed to analyze the dynamic changes in the MA.

## Conclusions

Our findings suggest that some patients with AF will develop moderate to severe MR (atrial functional MR). The mechanism of atrial functional MR involves multiple factors; in particular, MA dilatation, flattening of the annular saddle shape and leaflet tethering are the most influential factors affecting deterioration.

## Abbreviations

3D-TEE: 3 dimensional transesophageal echocardiography
AF: Atrial fibrillation
AH: Annular height
AHCWR: Annular height to commissural width ratio
ANOVA: Analysis of variance
APD: Anteroposterior diameter
CW: Commissural width
ERO: Effective regurgitation orifice
EROA: Effective regurgitation orifice area
LA: Left atrium
LAAPD: Left atrial anterior posterior diameter
LV: Left ventricle
LVEDD: Left ventricular end diastolic diameter
LVEF: Left ventricular ejection fraction
LVESD: Left ventricular end systolic diameter
MA: Mitral annulus
MR: Mitral regurgitation
MV: Mitral valve
PM: Papillary muscle
RJA: Regurgitation jet area
TTE: Transthoracic echocardiography
